# Peruvian Maca (*Lepidium peruvianum*) – III: The Effects of Cultivation Altitude on Phytochemical and Genetic Differences in the Four Prime Maca Phenotypes

**Published:** 2017-06

**Authors:** Henry O. Meissner, Alina Mscisz, Marek Baraniak, Ewa Piatkowska, Pawel Pisulewski, Mieczyslaw Mrozikiewicz, Teresa Bobkiewicz-Kozlowska

**Affiliations:** 1 Faculty of Health Studies, Charles Sturt University & Therapeutic Research, TTD International Pty Ltd, 39 Leopard Ave., Elanora, QLD 4221, Australia;; 2 Research Institute of Medicinal Plants, 27 Libelta St., 61-707 Poznan, Poland;; 3 Faculty of Food Technology, Cracow University of Agriculture, 122 Balicka St., 30-149 Krakow, Poland;; 4 Department of Pharmacology, Poznan University of Medical Sciences, Rokietnicka 5a, 60-806 Poznan, Polanda

**Keywords:** Altitudes, Four Phenotypes, Hypocotyls, Lepidium peruvianum, Maca, Phytochemistry

## Abstract

In two trials, dietary and Glucosinolates’ characteristics in four Maca phenotypes have been examined with an extension into the determination of DNA sequences. Hypocotyls of the four prime phenotypes of Peruvian Maca - *Lepidium peruvianum* Chacon, labelled as “Yellow”, “Black”, “Red” and “Purple” were separated from mixed Maca crops cultivated in four geographically-distant locations in the Peruvian Andes at altitudes between 2,800m and 4,300 m a.s.l. It was found that at higher altitudes where Red and Purple Maca phenotypes were grown, the significantly higher (*P*<0.05) Glucosinolates’ concentrations, adopted as the marker of Maca physiological activity, were observed with the Purple phenotype showing the highest Glucosinolates’ content at 4,300m a.s.l., followed by the Red-coloured hypocotyls. Black Maca showed a reversal, but also a significant (*P*<0.05) trend, while the Yellow phenotype showed no visible altitude-inflicted response (*P*>0.05) and has consistently the lowest Glucosinolates content. Thus, it is reasonable to assume that the altitude at which Red, Purple and Black phenotypes of *L. peruvianum* are grown, may be responsible for the variation in physiologic functionalities, leading to different than expected specific therapeutic and health benefits induced by Maca phenotypes grown at diverse altitudes. Although promising, insufficiently precise differences in DNA sequences failed to distinguish, without any reasonable doubt, four Maca phenotypes cultivated either in the same or geographically-distant locations, and harvested at different altitudes a.s.l. Further research on DNA sequences is needed, with more primers and larger number of Maca phenotypes, considering biosynthesis of secondary metabolites and adaptation pathways induced by harsh environment at altitudes where Maca is cultivated.

## INTRODUCTION

Since the time of the Incas, Peruvian Maca (*Lepidium peruvianum* Chacon), has been traditionally used by indigenous Peruvians as a medicinal herb and a vital staple food component (1-5). Peruvian Maca is a unique Andean *Lepidium* species with a disomic octoploid genome (2n = 8× = 64) (4) which has been cultivated under environmental conditions peculiar to the hostile habitat existing in the high Peruvian Andes (6, 7), mostly above 4000m above sea level (a.s.l.). Traditionally acknowledged therapeutic properties have been described by Gonzales (5, 8) and Meissner *at al.* in previous papers from this series (9-12).

There are strong indications (7, 8, 13) that the therapeutic properties of Peruvian Maca and its fertility-enhancing properties in particular, may be linked to high concentrations of Glucosinolates (benzyl and p-methoxybenzyl glucosinolate and their isothiocyanate derivatives), which represent one of the major functional groups of biologically active compounds in this herb (7, 13, 14). It has been further observed that several Glucosinolates’ concentrations determined in hypocotyls of various coloured Maca phenotypes (8, 15-17), may be linked to specific gender-and age-dependent physiological and therapeutic properties induced by oral administration of Maca preparations, as demonstrated in a series of studies on laboratory animals (18-23), and on humans using both male subjects (24) and pre- and post-menopausal women (9-12). It has been further found that the Maca phenotype characterised by the colour Red, has a selective therapeutic functionality affecting men after 50, with a capacity to prevent and reduce prostate hyperplasia (21-23). Black Maca phenotype was found as having a positive effect on sexual desire, aphrodisiac and fertility-enhancing properties in adult healthy men (5, 24, 25). On the other hand, Yellow based Peruvian Maca in a mix with other phenotypes in a traditionally-harvested Maca crop, had a positive hormone-balancing effect on pre- and post-menopausal women (10-12) thereby showing potential as a non-hormonal alternative to hormone replacement therapy (HRT) treatment.

In an earlier laboratory study (16, 17), four major Maca phenotypes identified by the colour of hypocotyls “Yellow”, “Red”, “Black” and “Purple”, demonstrated the existence of substantial differences in determined yields, size and weight of hypocotyls, as well as in Glucosinolates at both levels and chromatographic profiles. Irrespective of the cultivation in geographically-distant locations in the high Andes at about 4,200 m a.s.l., the highest content of Total Glucosinolates was recorded in the Red Maca phenotype, followed by Black and Purple, with the Yellow phenotype showing consistently lower glucosinolates levels (17). Also, there were indications that DNA sequences and related genetic profiles can potentially to be developed as a useful tool in identifying individual Maca phenotypes (16).

From the results presented in the previous papers within this series (16, 17) it could be assumed that Peruvian Maca phenotypes grown at the same altitude but in geographically-distant cultivation locations, are most likely exposed to different environmental conditions. This may significantly influence morphology (size and weight) of harvested hypocotyls and the resultant biochemical profiles of Maca phenotypes. Such an assumption has been supported by studies conducted in the Himalayas and the European Alps (26, 27), demonstrating that environmental conditions at different locations, linked to the altitude at which mountainous medicinal plants were grown, are responsible for their phytochemical characteristics. Substantial differences existing in both yields and corresponding chromatographic profiles of Glucosinolates, determined in the Yellow, Red and Black Maca hypocotyls collected from various cultivation locations in the Peruvian highlands, were also reported by Gonzales (8). However, phenotypic differences in phytochemical profiles observed in the Maca phenotypes as reported inall the above study, could be compounded by agro-environmental factors associated with the geographic location where Maca was cultivated.

Therefore, in this paper, an attempt has been made to determine to what degree the altitude at which Peruvian Maca is cultivated may induce change - if any - to phytochemical characteristics, Glucosinolates’ contents and genetic profiles of the four main colours of hypocotyls considered as major Maca phenotypes grown at three altitudes. These Maca cultivation sites were either positioned in three different geographically-distant locations, or on a single plantation site where Maca crops were cultivated at three different altitudes on a mountain slope in high Peruvian Andes.

## MATERIAL AND METHODS

Peruvian Maca (*L. peruvianum* Chacon): The plant species was phytochemically characterised in the previous papers from this series (15-17).

In two separate Trials, several samples of hypocotyls representing Maca phenotypes “Yellow”, “Purple”, “Black” and “Red” were separated from mixed harvested Maca crops cultivated in locations situated in the highlands of the Peruvian Andes at diverse known altitudes above sea level (a.s.l.).

**Figure 1:**
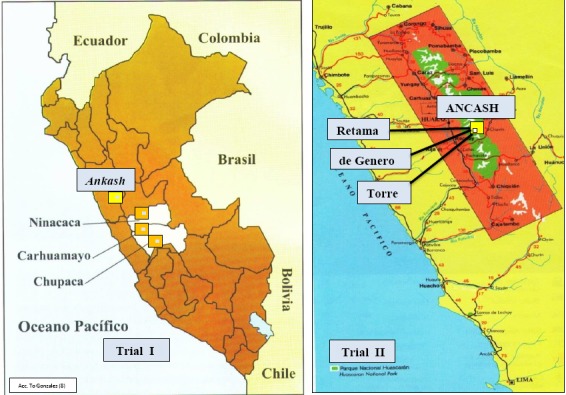
Indication of Peruvian Maca cultivation areas located in Andean Highlands used in the study, with three different altitudes where Maca was grown (metres above sea level - m a.s.l.) identified as: in Trial I: Three geographically-distant locations in High Andes where Maca was cultivated: Chupaca (Yanacancha 3,800 m a.s.l.), Curhamayo (4,000 m a.s.l.), Ninacaca (4,200 m a.s.l.) and in Trial II: Three Maca planting sites in one cultivation location on a mountain slope in Ancash at three altitudes: Torre (4,000 m a.s.l.), de Genero (4,150 m a.s.l.) and Retama (4,300 m a.s.l.).

**Figure 2:**
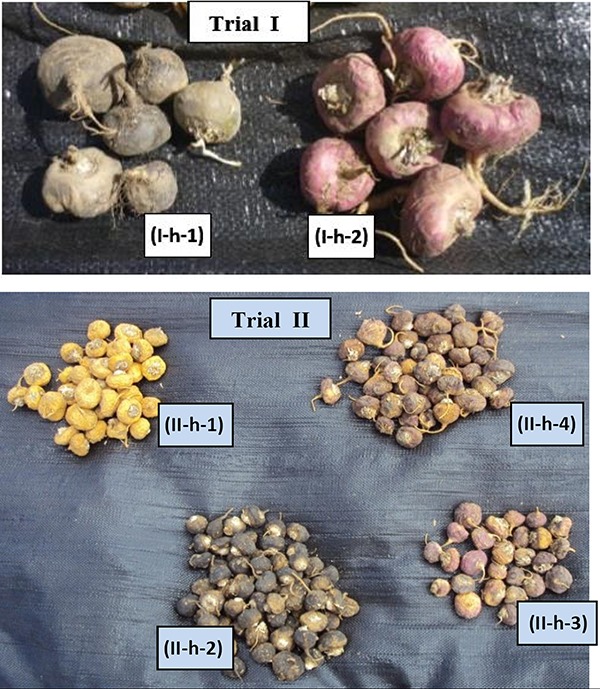
Representative samples of the prime two (Trial I) and four (Trial II) Peruvian Maca phenotypes represented by colours of hypocotyls separated from traditionally-harvested mixed Maca crop cultivated in Andean Highlands. Depicted are hypocotyls of Maca phenotypes at initial stages of an "openair drying" process, when four prime Maca phenotypes can be easily distinguished and separated from the freshly-harvested mixed Peruvian Maca crop on the basis of hypocotyl colour: Trial I: Red (I-h-1) and Black (I-h-2) and in Trial II: Yellow (IIh-1), Black (II-h-2), Purple (II-h-3) and Red (II h-4).

Trial I: Samples of Peruvian Maca hypocotyls (*Lepidium peruvianum*) representing “Black” and “Red” phenotypes were collected from three altitudes 3,800 m., 4,000 m. and 4,200 m. a.s.l. located at geographically-distant cultivation sites in the altitudinous Andes based in Yanacancha (Chupaca), Carhuamayo (Junin) and Ninacaca (Pasco) respectively (Figure 1; Trial I). Samples of hypocotyls were collected according to the procedure described earlier (16). Sub-samples of the intact dry hypocotyls of both phenotypes (Figure 2) were sent to three Laboratories (labelled A; B and C) for analysis of Glucosinolates (both concentrations and HPLC spectra) according to procedures described previously (16). Basic nutritional characteristics (proximate analysis and Fatty Acids) were also determined in Laboratory A using procedures described earlier (17). The HPLC resolutions for the two Maca phenotypes, Red and Black grown at three altitudes, were extended in Laboratory C into 3D Maca spheroids (16), for possible use as a “fingerprint” to assist in distinguishing the two Maca phenotypes.

**Figure 3:**
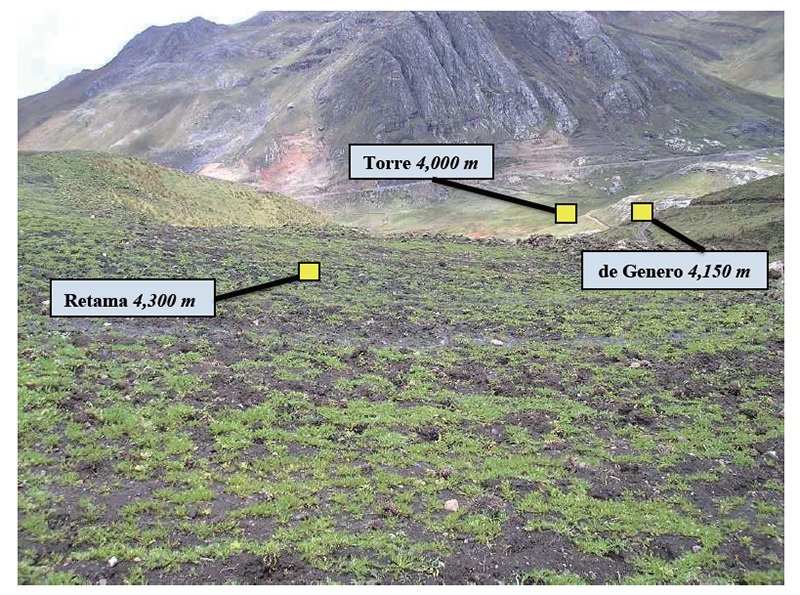
Trial II: A view of Ancash Maca cultivation location (viewing from the highest cultivation site down to the lowest) in High Andes (Cordilleras Blanca) at three altitudes (metres above sea level - m a.s.l.) w here Maca was grown, identified as: Torre (4,000 m a.s.l.), de Genero (4,150 m a.s.l.) and Retama (4,300 m a.s.l.).

Trial II: Glucosinolates levels in four Peruvian Maca phenotypes grown at three altitudes in Ancash: Four main phenotypes of Peruvian Maca *(L. peruvianum)* identified by the colour of hypocotyls “Yellow”, “Purple”, “Black” and “Red” (Figure 2: Trial II), were obtained from a single Maca plantation located on the western slopes of the Cordillera Blanca mountain range (18L, 271,129 E, 8'910,105 S) in Ancash (Figure 1; Trial II) at three cultivation altitudes: 4,000 m - “Torre”, 4,150m - “De Genaro” and 4,300 m a.s.l. - “Retama” (Figure 3). Morphologic diversity of the four Maca phenotypes cultivated in Ancash has been described previously for the crop grown at 4,150 m a.s.l. (17), and characterized by typical percentage distribution of hypocotyls in a mixed Maca crop recorded in this location: “Yellow” – 61.1%, “Black” - 6.8%, “Purple” – 22.2% and “Red” – 9.9%. The Ancash plantation site together with the three Maca cultivation altitudes was attested for organic status SKAL (28).

Traditionally-dried hypocotyls (up to two months an open-air drying at harvesting site) of the four Maca phenotypes were sent to Laboratory B for Glucosinolates’ analysis, according to the procedure described earlier (16).

The data obtained from proximate analysis, Fatty Acids and Glucosinolates determinations were presented as means ± SD (n=2). One-way, parametric analysis of variance (Statistica v. 8.1, StatSoft, Inc., Tulsa, OK, USA) was applied for testing the differences between the experimental treatments. The Duncan’s test was used for the identification of statistically significant differences at a level of p<0.05.

### Genetic variability

Analysis of Genetic variability in four Peruvian Maca (*L. peruvianum* Chacon) phenotypes: Black (B), Red (R), Yellow (Y) and Purple (P) grown at three altitudes, were completed according to the procedure described in one of the previous papers from this series (16). In order to observe trends in changes of DNA profiles related to the four Maca phenotypes and the effect of the altitude where they were grown, either in a single plantation or in geographically-distant locations under different set of environmental conditions and soil type, genetic sequencing and the comparison of DNA profiles were conducted within and between samples grouped in five alternative blocks arranged as follows:

- extracting DNA from freshly harvested Maca hypocotyls cultivated and collected as Fresh at the Ancash location – ANCASH Fresh at two altitudes: 4,000 m (1) and 4,300m (3) above sea level (a.s.l.) and classified in commercial terms as Small (17);
- extracting DNA from traditionally-dried Maca hypocotyls cultivated at the Junin location at two altitudes: 4,000 m and 4,200 m a.s.l. and separated into a hypocotyl size/weight class defined previously for Junin crop as Small (17);
- the same as (b) above but the hypocotyl size/weight class being defined as Large;
- DNA extracted from traditionally-dried Red Maca hypocotyls collected in three geographically-distant locations (G-D-L) at altitudes 3,800 m (R38), 4,000 m (R40) and 4,200 m a.s.l. (R42), and
- DNA from the four Dry Maca phenotypes collected at the Ancash location, Small hypocotyls (ANC-S) cultivated at the altitude 4,300 m a.s.l., provided a control to the same phenotype samples analysed as fresh in (a).

Prior to the complex comparison of the four phenotypes grown at three altitudes, three Maca phenotypes Black, Red and Yellow, previously studied (16), were tested as a cross-reference to the series of DNA comparisons attempted in this paper. Two selected primers (OPL-12 and OPL-13), previously shown, were used to provide clear and distinct resolution of DNA bands in the phenotypes of L. peruvianum: Lb = Black; Lcz = Red; Lz = Yellow.

In all genetic sequencing of Maca phenotypes presented in this paper, genotypic characteristics were determined by means of the ISSR-PCR (Inter-Simple Sequence Repeats polymerase chain reaction) and RAPD (Randomly Amplified Polymorphic DNA) techniques. In the genetic sequencing tests 50 ng/µl quantity of isolated DNA from all tested hypocotyl samples was applied in PRD reactions with 20 pmol of each primer (OPL Kit OPERON or single RAPD primer OLIGO) and 7.5 μl of PCR Mix (Fermentas 2×). The final volume of the reactions was 15 μl. Sequencing was done with the use of 20 RAPD primers (Operon Tech., kit L) and 17 ISSR primers with a majority of 3’ anchoring sequence. PCR-RAPD and PCR-ISSR reactions formed the basis of testing genetic profiles in analysed Maca samples. Results of reactions were primarily tested on 1.5% agarose gel and then on polyacrylamide gel with silver staining.

## RESULTS

### Trial I. Phytochemical characteristics of two Peruvian Maca phenotypes collected from three altitudes in different geographically-distant locations

The two Peruvian Maca *(L. peruvianum)* phenotypes Red and Black collected in three Maca planting sites Chupaca (Yanacancha 3,800 m a.s.l.), Curhamayo (4,000 m a.s.l.), Ninacaca (4,200 m a.s.l.) were analysed as dried hypocotyls. With the exception that the 4000 m a.s.l., Red Maca contained statistically higher Crude Protein levels (*P*<0.05) when compared to Black Maca grown at the same altitudes (Table 1). There was a trend that at higher elevations the mineral (Ash) content in both Maca phenotypes was substantially higher than in Maca grown at lower altitudes (*P*<0.05). The opposite trend was evident in contents of Crude Fat, which decreased with higher altitude at which both Maca Phenotypes were cultivated (*P*<0.05). This observation led to the analysis of individual Fatty Acids, profiles of which are presented in Table 2 (as single determinations only).

**Table 1:** Trial I: Nutritional composition of two Peruvian Maca phenotypes (*L. peruvianum*) distinguished by the colour of hypocotyls: Red and Black, cultivated at three different altitudes (3,800 m, 4,000 m and 4,200 m above sea level (a.s.l.))^1^

Maca Phenotype and meters above sea level	Dry Matter (g/ 100 g)	Crude Protein (Nx6.25) (g/ 100 g)	Crude Fat (g/ 100 g)	Ash (g/ 100 g)	Carbo-hydrates (g/ 100 g)	Fibre (g/ 100 g)

**Red 3,800 m**	83.8 ± 5.5^a^	18.6 ± 0.1^c^	1.2 ± 0.0^b^	3.7 ± 0.0^a^	76.6 ± 0.0^a^	4.5 ± 0.1^b^
**Red 4,000 m**	88.1 ± 0.0^a^	10.1 ± 0.1^a^	0.4 ± 0.1^a^	4.6 ± 0.0^b^	84.9 ± 0.3^c^	12.2 ± 0.2^c^
**Red 4,200 m**	86.7 ± 1.0^a^	17.3 ± 1.5^c^	0.4 ± 0.0^a^	5.1 ± 0.0^d^	77.2 ± 0.3^a^	3.0 ± 0.7^a^
**Black 4,000 m**	89.2 ± 0.1^a^	8.8 ± 0.4^a^	1.0 ± 0.1^b^	4.7 ± 0.0^b^	85.6 ± 0.3^d^	4.8 ± 0.1^b^
**Black 4,200 m**	85.8 ± 0.4^a^	12.5 ± 0.3^b^	0.2 ± 0.1^a^	5.0 ± 0.1^c^	82.3 ± 0.3^b^	9.2 ± 0.8^c^

1 Mean values shown in columns and denoted by different superscript letters are statistically significant at p<0.05 level.

**Table 2:** Trial I: Profiles of Fatty Acids (g·mol−1) in the two phenotypes of Peruvian Maca (*L. peruvianum*) distinguished by the colour of hypocotyls: Red and Black cultivated at three different altitudes (m a.s.l.)^1^

C-length	Fatty Acid	Red 3800	Red 4000	Red 4200	Black 4000	Black 4200

C14:0	Tetradecanoic (myristic)	1.44	1.29	0.42	0.3	0.22
C15:0	Pentadecanoic	0.19	0.29	0.24	0.2	0.21
C16:0	Hexadeconic (palmitic)	23.6	34.39	31.6	27.81	26.1
C16:1	9-cis-Hexadecenoic (palmitoleic)	0.57	1.55	1.09	1.12	0.89
C17:0	Heptadecanoic (margaric)	0.34	0.5	0.42	0.44	0.32
C18:0	Octadecanoic (stearic)	2.42	3.37	2.93	3.08	2.59
C18:1	(E)-octadec-9-enoic (elaidic)	10.48	15.31	13.57	12.34	13.87
C18:2	9, 12-octadecadienoic (linoleic)	38.12	23.36	27.74	30.44	31.67
C20:0	Icosanoic (arachidic)	0.47	0.46	0.51	0.43	0.5
C18:3	9,12,15 Octadecatrienoic (α-Linolenic)	16.78	10.07	13.43	12.6	17.12
C22:0	Docosanoic (behenic)	0.53	0.5	0.53	0.3	0.43
C24:0	Tetracosanoic (lignoceric)	n.d.^2^	0.25	0.26	0.33	0.32
C24:1	(Z)-Tetracos-15-enoic (nervonic)	n.d.^2^	0.28	0.2	0.22	0.28

1 Single determination only;

2 n.d.= Not detected.

Higher elevation substantially lowered the contents of Myristic Acid in the Red Maca phenotype. Those concentrations were much higher than detected in the Black Maca phenotype grown at similar altitudes, while at higher altitudes, Palmitic, Palmitoleic and Elaidic Acids were visibly at higher concentrations than in Maca hypocotyls grown at lower altitudes with both Linoleic and α-Linolenic Acids showed a reversed trend. There were no substantial differences within concentrations of Fatty Acids in Black Maca grown at different altitudes, except for the α-Linolenic Acid content, which was substantially higher at the higher altitude. The reverse trend was observed in the Red Phenotype.

Irrespective of the method used in the three laboratories where analyses were performed, in each case, distinctively higher Glucosinolates’ concentrations (*P*<0.05) were detected within the hypocotyls of Red phenotypes when compared to the Black Maca specimens (Table 3). Concentrations determined in Red Maca phenotypes significantly increased (*P*<0.05) with the higher altitude where Maca was grown.

**Table 3:** Trial I: Glucosinolates concentration (g%) in two Peruvian Maca phenotypes (*L. peruvianum*) Red and Black: hypocotyls collected in three location at the altitudes 3,800 m, 4,000 m and 4,200 m a.s.l. as analysed in three Laboratories using three analytical methods*

Peruvian Maca Phenotype	Laboratory	3,800 m Chupaca	4,000 m Curhamayo	4,200 m Ninacaca	± SD**

**Red**	**A** 1	0.058 a I	0.584 b I	0.993 c I	± 0.241
	**B** 2	0.183 a II	0.484 ab I	0.511 b I	
	**C** 3	0.201	0.219	0.378	
**Black**	**A**	n.d.	0.1 a I	0.08 a I	± 0.063
	**B**	n.d.	0.272 b I	0.08 a I	
	**C** 3	n.d.	0.177	0.088	

* Values in each row with unlike lower case letters indicate an existence of statistically significant differences at *P*<0.05 level in Glucosinolates contents within each Laboratory between three altitudes where Red and Black Maca phenotypes were grown while unlike Roman numbers within the column in each phenotype indicate significant differences between results obtained by the use of different methods used for analysis of Maca phenotypes in Laboratory A and B only at *P*<0.05 level.

** ± SD, Standard Deviation of mean; n.d., not determined (a sample was not obtained).

1 Faculty of Food Technology University of Agriculture Cracow, Poland (Adoption of the method by Michalski *et al.* (1995) and Kraling *at al*. (1990) - determined against glucotropaeolin standard);

2 Research Institute of medicinal Plants Poznan, Poland (Adoption of the method by Li *et al.* (2001) - determined against glucotropaeolin standard);

3 Analytical Division, Plant Science, SCU, Lismore, Australia (Using the method by Piacante *et al.* (2002) and Mc Lure (2004)-determined against Sinigrin standard). *Statistic not available due to single determination supplied only*.

Contrary to the Red Maca, irrespective of the method used, Glucosinolates’ concentrations in the Black phenotype, decreased with altitude. Results obtained in Laboratory B confirmed a statistically significant decrease (*P*<0.05) in values reported between 4,000m and 4,200m a.s.l. Laboratory C has not provided sufficient data for statistical analysis, although the reported results were closer to the values obtained by Laboratory B for both phenotypes.

The method used in Laboratory A (28 and 29) provided higher Glucosinolates results as compared to methods used in Laboratory B (13) and C (30 and 31). However, only the results reported for Red Maca grown at 3,800 m a.s.l. (Table 3) were confirmed as statistically different (*P*<0.05).

### 3D spectra

**Figure 4:**
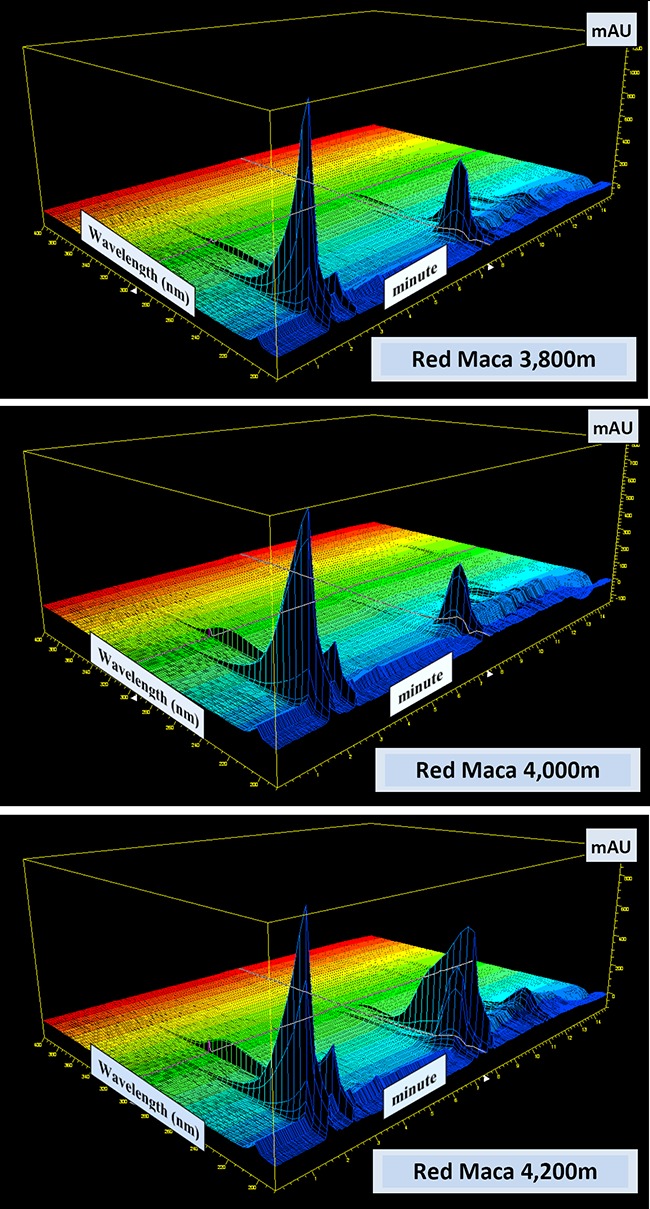
3D HPLC Resolutions: Peruvian Maca (L. peruvianum) phenotypes "Red" grown at altitude 3,800m; 4,000m and 4,200m a.s.l. Chromatographic profiles were generated with the use of 3D plot mode of HP Chemstation software from analysis of Glucosinolates (against Sinigrin standard) selected as an active compound in cultivated Peruvian Maca. [Glucosinolates determined in Red Maca: at 3,800m = 0.201%, 4,000m a.s.l. = 0.219% and at 4,200m a.s.l. = 0.378%].

**Figure 5:**
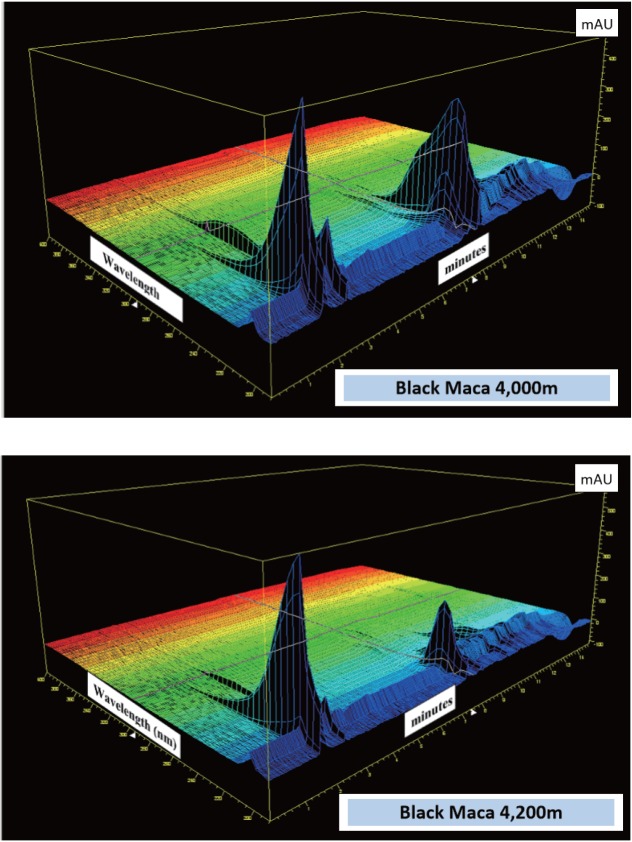
3D HPLC Resolutions: Maca (L. peruvianum) phenotypes “Black” grown at altitude 4,000 and 4,200m a.s.l. Chromatographic profiles were generated with the use of 3D plot mode of HP Chemstation software from analysis of Glucosinolates (against Sinigrin standard) selected as an active compound in cultivated Peruvian Maca. [Glucosinolates determined in Black Maca at 4,000m a.s.l. = 0.177% and at 4,200m a.s.l. = 0.088%].

There were distinctive differences in the 3D spectra mode of HP Chemstation software between the Red Maca phenotypes grown at three altitudes 3,800 m, 4,000 m and 4,200 m a.s.l. with noticeable differences in areas of scanned Glucosinolates and derivatives visible on the HPLC resolution chart between 2 – 3 min and 8 - 9 min (Figure 4). In the Black Maca phenotype, the pronounced differences were noted in the areas of spectra representing Glucosinolates and derivatives detected between 2 – 3.5 min and 9 to 10 min on the 3D HPLC chart (Figure 5) for two altitudes 4,000 m and 4,200 m a.s.l.. There were many unidentified peaks on the HPLC resolution chart. Standards were not available in the Laboratory C at the time when this work was conducted. Statistic comparison of HPLC charts and 3D spectra applying available packages already developed for such a purpose will be worth further investigation.

### Trial II. Glucosinolates in Peruvian Maca phenotypes cultivated in one plantation at three altitudes

Maca *(L. peruvianum)* phenotype Red grown at the altitude 4,000 m a.s.l. contained statistically lower (*P*<0.05) Glucosinolates’ levels as compared to Black, Purple and Yellow hypocotyls with Black hypocotyls demonstrating the highest Glucosinolates values at this altitude (Table 4). Reverse Glucosinolates patterns was observed at the altitude 4,300 m a.s.l. where Red together with the Purple Maca showed significantly higher Glucosinolates’ concentrations (*P*<0.05) as compared to the Black and Yellow Maca phenotypes. The lowest values of the all four phenotypes was recorded in the Black Maca. There were no statistically significant differences (*P*>0.05) observed in Glucosinolates’ content between the four Maca phenotypes cultivated at 4,150 m a.s.l.. There was no significant (*P*>0.05) change detected in Glucosinolates’ content in the Yellow Maca phenotype which could be linked to the altitude where this phenotype was cultivated.

**Table 4:** Trial II: Glucosinolates concentration (g%)^1^ in hypocotyls of the four Peruvian Maca phenotypes (*L. peruvianum*) Red, Black, Purple and Yellow cultivated and collected in a single location on the slope of Ancash plantation site at the altitudes 4,000 m, 4,150 m and 4,300 m a.s.l.*

Maca Phenotype	4,000 m *Torre*	4,150 m *De Genaro*	4,300 m *Retama*	± SD

Red	0. 278 a I	0.512 b I	0.926 c I	± 0.233
Black	0.622 a II	0.418 ab I	0.114 b II	± 0.201
Purple	0.486 a I II	0.693 a I	1.136 b I	± 0.224
Yellow	0.418 a I II	0.471 a I	0.522 a III	± 0.067

* Values within the rows with unlike lower case letters indicate an existence of statistically significant differences at *P*<0.05 level between hypocotyls within each of the four Maca phenotypes cultivated at altitudes 4,000 m, 4,150 m and 4,300 m above sea level, while unlike Roman numbers within each column indicate significant differences (*P*<0.05) between results obtained from analysis of Red, Black, Yellow and Purple hypocotyls of Peruvian Maca phenotypes.

1 Results obtained in Laboratory B with the use of the method by Li *et al.* (2001) and determined against Glucotropaeolin as an external standard.

### Assessment of genetic variability in Maca Phenotypes cultivated at three altitudes

Prior to the complex comparison of the four phenotypes grown at three altitudes, on one occasion, three Maca phenotypes Black, Red and Yellow, previously studied (16) had been tested as a cross-reference to the series of DNA comparisons attempted in this paper.

**Figure 6:**
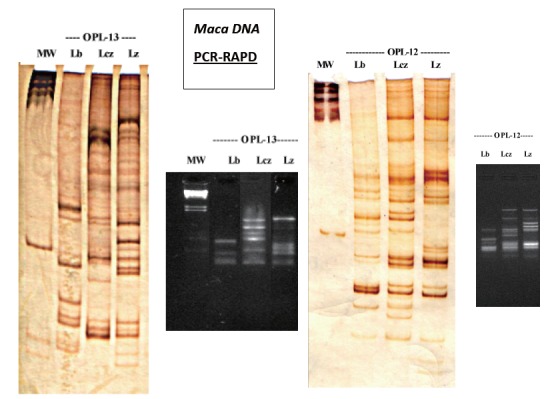
Trial II: DNA extracted from three Peruvian Maca hypocotyls (L. peruvianum Chacon) grown at 4,300m a.s.l. and processed in their fresh state: Lb = Black; Lcz = Red; Lz = Yellow. Profiles of lines observed in OPL-RAPD analysis may indicate an existence of genetic polymorphism in three analysed Maca phenotypes. ^*^Abbreviations above each of the three phenotypes sequence (Lb, Lcz and Lz) indicate wells and corresponding primer code used for the OPL sequence. The reaction used: 50 ng DNA, 20 pmol of each primer (OPL Kit OPERON or single RAPD primer OLIGO), and 7.5 μl of PCR Mix (Fermentas 2x). The final volume of the reactions was 15 μl.

Two selected primers (OPL-12 and OPL-13), previously shown as providing clear and distinct resolution of DNA bands in Maca, were used in preliminary DNA sequencing of three phenotypes of *L. peruvianum*: Lb = Black; Lcz = Red; Lz = Yellow (Figure 6). These phenotypes originated from the cultivation site in Junin at 4,200m a.s.l. and had been processed after traditional drying at the cultivation site. The results obtained from the identification of DNA sequences in the three tested Peruvian Maca phenotypes indicate an existence of distinctive sequence patterns which differentiate compared Maca phenotypes. Presence of polymorphism was clearly visible on both agarose and polyacrylamide gel with silver staining (Figure 6). This allowed for genetic differentiation based on the distinctive difference in DNA sequences represented by the three tested Maca phenotypes.

**Figure 7:**
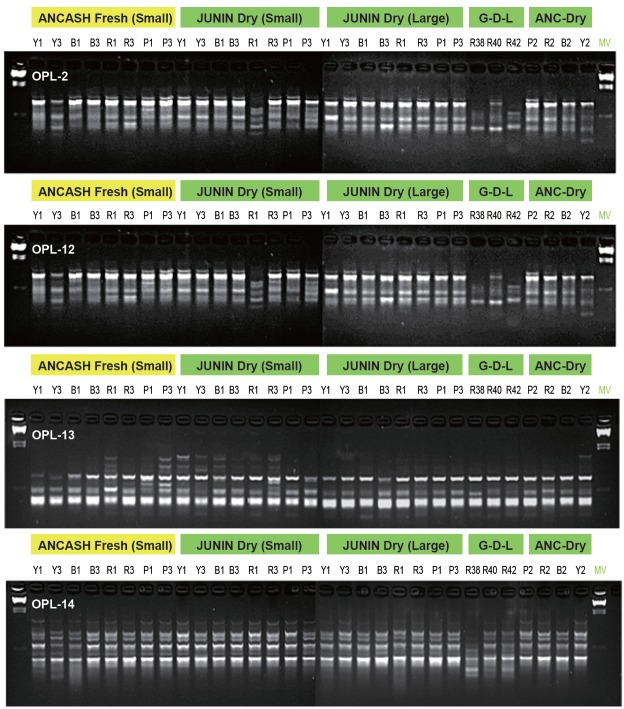
DNA extracted from hypocotyls of the four Maca phenotypes (L. peruvianum Chacon)* processed: B = Black; R = Red; Y = Yellow and P= Purple cultivated and collected as Fresh in Ancash location at two altitudes: 1 = 4,000 m and 4,300m above sea level (a.s.l.) compared with hypocotyls collected as Dry in Junin at: 1= 4,000 m and 3 = 4,200 m (as Small and Large hypocotyls) and for Red Maca (R) collected as dry in three geographically-distant locations = G-D-L at R38 = 3,800 m, R40 = 4,000 m and R42 = 4,200 m a.s.l. and Control Four Maca phenotypes collected as Dry in Ancash location at 4,300m a.s.l.. Different profiles in resolution bands observed in RAPD analysis, may indicate an existence of genetic polymorphism within and between series of compared samples with the four selected primers (OPL-2; OPL-12; OPL-13 & OPL-14). ^*^Boxes above each of the DNA sequence resolutions for the four Maca phenotypes (or three for the G-D-L Red phenotype only) represent resolutions related to individual phenotype cultivated at the specific altitude a.s.l. with the use of individual primer code displayed at the left top DNA resolution picture. The reaction used: 50 ng DNA, 20 pmol of each primer (OPL Kit OPERON or single RAPD primer OLIGO), and 7.5 μl of PCR Mix (Fermentas 2x). The final volume of the reactions was 15 μl.

In the main comparative DNA study (Figure 7), a total of 31 Maca samples were grouped into five sequence blocks consisting of four phenotypes: B = Black; R = Red; Y = Yellow and P= Purple (Figure 2; Trial II). Each phenotype was selected from the Maca crop grown at two altitudes between 4,000m to 4,300 m a.s.l. and processed as Fresh or Dry. Out of twenty commercial OPL primers used in the PCR-RAPD reactions, the four, giving the most distinctive resolution of DNA bands: OPL-2, OPL-12, OPL-13 and OPL-14, were selected for analysis of DNA profiles.

Differences in DNA profiles as represented by resolution bands within and between the series of compared blocks of Maca samples across the four selected primers, may indicate an existence of genetic polymorphism. However, when analysing Ancash samples from which DNA was extracted in a Fresh or Dry form, it was apparent that DNA extracted from “Fresh” hypocotyls showed much clearer phenotypical differentiations in the DNA profiles. This was particularly visible when comparing the DNA spectra of Yellow and Black phenotypes (Figure 7). Differences in DNA profiles observed between contrastive Maca phenotypes at three various altitudes, most likely depend on spontaneous deletions or insertions resulting in polymorphisms between them.

## DISCUSSION

Back in 1961 when Chacon (32) re-discovered Peruvian Maca - a nearly extinct and important ancient Inca sacral and medicinal herb, she identified several biologically active constituents within the tuberous part of this plant. Using laboratory tools available at that time, the active constituents were broadly grouped into seven categories: Alkaloids, Saponins, Tannins, Sugars, Anthocyanins, Starch and Fatty acids. Chacon (1) linked the function of these constituents to the traditionally-acknowledged nutritional and medicinal properties of this plant and maintained by native Peruvians until today. However she has not experimentally substantiated relative or direct target physiological effects of those Maca constituents in subsequent clinical research. In 1981 Johns (7) described another group of active compounds in Peruvian Maca represented by the aromatic Glucosinolates (benzyl and p-methoxybenzyl Glucosinolates in particular) and their isothiocyanate derivatives which are present in high concentrations in the edible underground part of this plant - hypocotyls. He indicated the existence of a possible link between the presence of Glucosinolates and the fertility-enhancing properties of Peruvian Maca.

With the use of currently available laboratory techniques, there are seven classes of distinctive secondary metabolites which are recognised as of physiological importance in Peruvian Maca (*Lepidium peruvianum);* these are: Glucosinolates (13), fatty acids (33), imidazole alkaloids (34), Amides (33, 35), Cyanogenic compounds (36), catechins (37) and Carbolines (30, 33). However, according to the survey conducted by Li *et al.* (13) on Maca hypocotyls and products based on *L. peruvianum*, the most abundant group of compounds detected in fresh and dry hypocotyls were Glucosinolates with the most prominent being aromatic Glucosinolates, Benzylglucosinolate (Glucotropaeolin) and p-methoxybenzylglucosinolate, thus confirming earlier analytical results reported by Johns (7). Also, Gonzales and his Research Group (8) noted that Maca hypocotyls contain relatively higher Glucosinolates level than is observed in other cruciferous crops such as white cabbage and cauliflower. Hence its potential use as a marker in screening Maca for its peculiar functionality and gender-related physiological effects. Gonzales (8) also stipulated that observed differences in Glucosinolates recorded in various phenotypes of Peruvian Maca may be linked to specific physiological and therapeutic properties of Maca phenotypes with possible influences on the physiological role of Maca as herb with traditionally acknowledged medicinal properties.

In the present study, with an increase in the altitude from 2,000 m to 2,300 m a.s.l. where Maca was cultivated there was a statistically significant gradual increase in Glucosinolates concentrations in both Red and Purple Maca phenotypes (*L. peruvianum*). The highest Glucosinolates concentration was reported in in the Purple Maca hypocotyls cultivated at the level 4,300 m a.s.l. The opposite pattern of changes to Red and Purple phenotypes was determined in the Black Maca phenotype where the lowest Glucosinolates level was detected at the 4,300 m. Interestingly enough, there was no statistically significant changes in Glucosinolates contents in the Yellow Maca phenotype linked to change in the altitude where this phenotype was cultivated. It is worth to note that Yellow hypocotyls represent a major proportion of the Maca crop (17).

According to Chandra (26), phenotypic - chemotypic variation in plant species is significantly influenced by environmental conditions where plants are grown, with the altitude being a significant determinant in plant morphology. Plants of the same species will often be smaller and have a different shape at higher altitudes. This has not been confirmed in relation to Peruvian Maca, since earlier study (17) demonstrated that the size of harvested hypocotyls was dependent more on agro-environmental factors which characterised individual Maca cultivation location above the altitude 4,000 m a.s.l. Similar to trend observed in the present study in Red and Purple Peruvian Maca phenotypes, investigated by Arroniz-Crespo. *et al* (38) chemotypic variation in aquatic liverworts (*Jungermannia exsertifolia*) collected along the elevation of mountain streams indicates an existence of direct correlation between an increase in altitude and the abundance of a group of secondary metabolites - phenolic caffeic acid derivatives (38). Another controlled study by Spitaler *et al.* (27), who looked at chemotypic variation in *Arnica Montana* grown from the same genetic stock and grown in trial plots at various altitudes in the Swiss Alps, shows a similar pattern of changes to the one observed on the Ancash plantation. This plantation had been established from the same genetic stock used in the Junin, using identical establishing, cultivation and harvesting practices as traditionally used in Junin plantations. The germplasm used by Spitaler *et al.* (27) was genetically controlled to allow for direct correlations between the environmental conditions and chemotypic variations. It was found that the proportion of flavonoids with hydroxylated B rings to flavonoids lacking this feature significantly increased with altitude. Also, amounts of a caffeoylquinic acid increased by more than 85% between the lowest and highest sites where plants were collected. This was similar to trend, but lesser in magnitude to changes observed in Glucosinolates’ content detected in Peruvian Red and Purple Maca phenotypes grown at 4,000 m and 4,300 m a.s.l., with 3.3-fold and 2.3-fold increase recorded for the Red and Purple phenotypes respectively. Interestingly enough, the Black Maca phenotype exhibited a reverse trend in Glucosinolates concentration, showing 5.4-fold decrease at higher cultivation altitude.

A common theme observed in the chemistry of compounds to increase their abundance in plants with altitude, is their inherent ability to absorb UV-B radiation. Exposure to UV-B radiation increases with altitude and is known to be a significant environmental factor affecting the growth of plants (39). UV-B radiation inhibits photosynthetic processes and can cause damage to DNA resulting in cell death. Plants have been observed to accumulate secondary metabolites in their leaves which protect them from the effects of UV-B radiation (40). Petrella *et al*. (41) reported that when rough bluegrass was exposed to constant high-intensity blue light, at intensities between 150 and 250 mmol·m-2·s-1, anthocyanin content in plants significantly increased and the white light resulted in an increased anthocyanin concentration (118-fold) compared to untreated plants, the situation which may be observed with an increase in altitude a.s.l.. Given that the benzyl Glucosinolates in hypocotyls of Red and Purple *L. peruvianum* are observed to increase with altitude – possibly to absorb UV-B radiation, it is feasible to assume that these secondary metabolites are produced as a defence mechanism against the higher levels of UV-B radiation which occur at increasing altitudes. Many plants produce secondary metabolites in their roots which are then distributed to the leaves. However, aerial parts were not examined in this study, so an existence of such a relationship cannot be confirmed at this stage. On the other hand, in Black Maca hypocotyls, Glucosinolates content decreased with the elevation, indicating that other mechanisms than response to UV-B radiation may control and be responsible for concentration of Glucosinolates, considered as the one of the main active therapeutic components in Peruvian Maca (7, 8).

Amongst the variety of secondary metabolite classes reported in Maca, a number of them have also absorbance maxima in the UV-B spectrum within the range 260 – 320 nm (Figure 4 and 5). These are octadecadienoic acids (33) and imidazolium alkaloids - Lepidilines (34), which have a strong absorbance maxima between 250 - 278nm (33, 34). UV maxima were not reported for macaridine, carbolines and amides. All have intermediate sized chromophores indicating that they are also likely to absorb in the UV-B range. This may also partially explain the strong opinion of Dr Chacon and Dr Obregon – Peruvian experts in Maca studies (personal communication – 2000, 17), that, for Maca displaying its optimal physiologic and therapeutic properties, the hypocotyls should be both cultivated and traditionally sun-dried on site of their propagation and harvest i.e. above 4,000m a.s.l.

Given the hypothesis that *L. peruvianum* accumulates higher concentrations of UV-B absorbing compounds with the altitude, it is also feasible to assume, that in addition to Glucosinolates, other classes of biochemically- and physiologically-active compounds present in Peruvian Maca may also increase in concentration and/or change absolute or relative ratios with the altitude at which Maca is cultivated. It has been reported that Peruvian Maca grown in the Andean highlands with the entire complex of active compounds, prevented ultraviolet A-, B-, and C-induced skin damage in rats, thus providing UV protection (42). Those compounds in the entire hypocotyl material, representing individual specific phenotypes may act in synergy, thus maximising physiological effects of the main group of functional Maca components – Glucosinolates, thereby inducing such properties as energising effects and the stimulation of gender-specific reproductive functions. These therapeutic properties had been confirmed in a number of clinical studies conducted on Peruvian Maca blends and individual phenotypes (9-12, 18, 20-25, 42-44).

As yet there is no clear understanding as to which precisely-defined chemical class or group of compounds, are actually responsible for the medicinal functionality of specific phenotypes of Peruvian Maca with their gender- and age-linked activity. Many of the reported compound classes in plants other that Maca have medicinal functionality such as Catechins from Green tea (*Camellia sinensis*) which are known for their high anti-oxidant value, Amides in *Echinacea* species which are associated with the immunomodulatory functions of this herb, Carbolines, containing well known MAO (monoaminooxidase) inhibitors having direct effect on hormone metabolism with various effects on sexual function in animals and Glucosinolates and their derivatives (isothiocyanates, sulphoraphane) in Maca and other *Brassica* species, which are generally acknowledged to be of therapeutic importance.

It is likely that there is an ecological link between the role of secondary metabolites in Maca as a plant defence mechanism (UV-B protection) and medicinal quality, given that some components in the plant are also associated with UV-B absorbance. In this study, it was observed that increasing Glucosinolates levels in Red and Purple Maca phenotypes with higher cultivation altitude may be one of a number of environmentally regulated chemical relationships in this species. The decrease observed in the Black Maca specimens with altitude may indicate a different biosynthetic response in this phenotype resulting in an increase in other related compounds and occurring changes in their absolute or relative ratios with altitude. A detailed chemotypic survey of Maca phenotypes across a range of ecological conditions has been provided in previous papers from this series (16, 17) with the further data on the topic expended in this paper. At some stage it will be essential to correlate phytochemical properties of Maca phenotypes to both traditional medicinal applications and currently-demonstrated selective therapeutic properties of Maca phenotypes established in clinical trials on men (5, 24, 25) and women (10-12). Such an empirical analysis would likely provide valuable insight into the medicinal functionality of Peruvian Maca, as well as identifying phytochemical Maca components as specific markers and actives characterising individual Maca phenotypes, which most likely would be responsible for the specific gender- and age-related physiological responses in humans ingesting specific Maca phenotypes of known origin in geographic (both latitude and altitude), environmental and agro-cultivation terms. This also would lead to higher degree of expectation in efficacy of the use of the proper Maca phenotype or their blends when assisting in treating the existing medical conditions and/or preventing their development.

An attempt to identify bioactive Maca components with a link to expected functionality and yet undefined metabolic targets of secondary Maca metabolites, namely, Maca alkaloids, Macaenes, and macamides, has been most recently presented by Fan Yi *et al.* (45). He applied a silico target fishing model in order to detect the relevance of individual Maca compounds to target their functions using already published research on Peruvian Maca. Fan Yi *et al.* (45) was able to identify significant metabolic effects of bioactive compounds and secondary metabolites present in Maca for the treatment and prevention of the following metabolic disorders: osteoporosis (8 targets), prostate cancer (9 targets), and kidney diseases (11 targets) as well as a new target determined in the adopted model - cardiovascular diseases (29 targets) - through distinctive function of Maca in reducing amide alkaloid level in blood vessels. In further work on Maca it will be worthwhile to apply the above model to individual Maca phenotypes to identify metabolic pathways involved in gender- and age- specific targets together with the functionality of bioactive components characterising individual Maca phenotypes.

### Effect of altitude on genetic diversity

DNA extracted from the three Maca phenotypes harvested in Junin (4,200m a.s.l.) indicate an existence of genetic polymorphism linked to specific profiles of DNA bands associated with three tested Maca phenotypes: Red, Black and Yellow, grown in the same location under identic environmental conditions (Figure 6). Irrespective of the primer used, each Maca phenotype had a distinctive profile of DNA which appeared phenotype specific, with the possibility to be used in the identification of specific phenotypes. In previously reported genetic study (16) using different primers, only the Yellow phenotype was clearly distinguished from the Black one, while with the use of the primer OPL-12 and OPL-13, each phenotype was clearly distinguished from their DNA profiles, thus indicating their possible use as a fingerprint identifying individual Maca phenotype grown at the same altitude a.s.l.

However no distinctive, both phenotype- nor cultivation-linked differences in DNA sequences were detected in four Maca phenotypes (Red, Black, Yellow and Purple) compared in Figure 7. This may indicate the existence of genetic diversity of Maca phenotypes grown under different environmental conditions at altitudes between 3,800 m and 4.300 m a.s.l. in a single or geographically-distant locations.

All the primers selected in this series of the main comparative study allowed for amplification of several segments, which could form specific genetic profiles of analysed phenotypes. However, the difference in the sequence of bands in DNA resolutions from Maca grown at different altitude were not distinctive enough to allow for identification of individual phenotypes, according to the commonality of single sections or entire sequences determined in individual phenotypes. DNA profiles from Yellow and Black phenotypes processed as fresh, displayed different band characteristics in Maca hypocotyls cultivated at altitude of 4,000 m and 4,300 m a.s.l. This may indicate that the altitude at which the Maca crop is cultivated may have a measurable genetic effect – at least in Yellow and Black phenotypes – as demonstrated in this (Figure 7) and in the previously reported study (16). DNA resolutions presented in Figure 7, provide also an indication that the Black and Red Maca hypocotyls may have close genotypical association.

Analysing all the DNA profiles compared in the main comparative study depicted in Figure 7, it is evident that the obtained sequences of bands in DNA profiles characterising individual Maca samples were inconsistent, making identification and distinction between samples far from being beyond reasonable doubt in regards to: (i) - the plantation where Maca was grown, (ii) - phenotype/colour of hypocotyl, (iii) - the size/weight of phenotype, nor (iv) - the altitude at which Maca was grown. Therefore, although the DNA profile techniques look promising in identification of phenotypes (Figure 6 and 7), in further research it will be essential to select a wider number and range of coloured Maca phenotype samples grown under different environmental conditions and conduct tests on wider range of primers. This would allow a link to be established between resultant specific genetic DNA profile representing the individual Peruvian Maca phenotype to specific therapeutic functionalities confirmed by the current biochemical knowledge and possible clinical study as scientifically-confirmed plant with corresponding target medicinal functions. Figure 7 demonstrates the basis of the above conclusion providing graphic representation of the outcome of DNA testing and justifying future DNA research on genetic diversity which appears to exist in Maca Phenotypes sourced from different cultivation locations.

The differences observed in Glucosinolates concentrations between the four Peruvian Maca (*L. peruvianum*) phenotypes (Red, Black, Yellow and Purple) grown at 4,000 m, 4,150 m and 4,300 m a.s.l (Table 4) and corresponding DNA profiles for the two extreme altitudes where the four Maca phenotypes were cultivated (Figure 7), could be due to various degrees of protein degradation in hypocotyls during dehydration process under the sun. At an elevation of 4,300 m a.s.l, UV light spectrum is more active than at lower altitudes at the same geographical location (i.e. 4,000m a.s.l.). Intensive UV and an extreme harsh environment may induce mutations of certain genes to help adapt to the environmental conditions. As reported by Quiros *et al.* (4), the mutations may involve the change between diploid an amphiploid status of the plant, thus increasing chances of adapting to the external conditions existing in the location where plant is forced to grow. Adaptation patterns studied on the example of *Podophyllum hexandrum* – an endangered medicinal plant grown in alpines of Central Himalaya (26) may confirm the above observation that there is a shift in a potential for the plant to survive in the environment at different altitudes, with different optimum adjusted to temperature for photosynthesis, and various conductance and transpiration rates.

From the results obtained in the present study, it would appear appropriate to base identification of Maca phenotypes on both Glucosinolates contents and/or the HPLC spectra, together with the corresponding DNA profiles with the use of selected most appropriate primers. This would allow for a clear DNA resolution relevant to the Maca phenotype and possibly – its origin. However, before suggesting adoption of this approach, more data needs to be examined in models with clear statistical limits, since in many cases, the associations determined with one evaluation model and scores plots are different when viewed with a different model.

With new tools and technologies constantly being developed, the growth of knowledge of the target metabolic functions of Peruvian Maca phenotype is increasing exponentially. An example could be a strong metabolomics evidence of Maca positively influencing cardiovascular conditions, confirming previously reported observation in clinical study (10, 11, 24, 25), through identifying for the first-time distinctive target function of Maca in reducing amide alkaloid level in blood vessels (45). Also, recent research-based clinical demonstration (46), show that in addition to a wide range of Maca target functions in alleviating pre- and post-menopausal symptoms (10-12), Maca (phenotype not specified) also appeared to be an effective treatment for antidepressant-induced sexual dysfunction (AISD) in women (46).

It appears that the DNA sequencing technique – once perfected and developed further – could be a valuable research and an important commercial tool in testing the authenticity of marketed Maca products based on a specific Maca phenotypes. This is because once Maca hypocotyls are powdered and incorporated into commercial marketable products (powder, tablets, capsules, etc.) the phenotype or genotype cannot be visually distinguished and since Maca phenotype which is identified by hypocotyl colour provides recognised by now different therapeutic functionality, is apparently linked to different market price, with the Black phenotype products demanding the highest price tag. Such a commercial reality opens possibility of product adulteration, for potential financial gains. Jin *et al.* (47) reported outcome of the analytical market survey demonstrating that materials labelled to contain powdered Maca root were partly or entirely substituted with turnip (*Brassica rapa*, Brassicaceae), radish (*Raphanus raphanistrum* subsp. *sativus*, syn. *R. sativus*, Brassicaceae), potato (*Solanum tuberosum*, Solanaceae), or corn (*Zea mays*, Poaceae). An investigation into the authenticity of Maca products distributed on markets in China with the use of DNA-barcoding approach indicates that adulteration of this currently popular herb although existing; it is not widespread (48). The same authors concluded, that the genetic variability was high enough to distinguish DNA Maca from the other species by comparing the ITS sequence of Maca in the GenBank database with ITS sequences of other species.

Jing Zhang1 (49) in a complex study on fresh mature leaves and tubers of a line of Maca plant (referred to as *Lepidium meyenii)*, cultivated for some 20 generations at altitude of 4,200 m in Yunnan province southwestern China, presented genomic basis for high altitude adaptation of this plant in the central Andes. The results of the above study showed that Genome of octoploid plant *Lepidium meyenii* (correct scientific name: *Lepidium peruvianum* Chacon – (15)) in the central Andes illuminates genomic basis for high altitude adaptation. Many Maca genes under strong positive selection were involved in the development and abiotic stress response, the trend which can be observed with an increase in the cultivation altitude. This may specifically apply to expansion of cold and UV-B adaptation pathways, hormone signalling and secondary metabolite biosynthesis pathways, which substantially strengthened the tolerance of Maca to harsh environment (49).

Jing Zhangi *et al.* (49) were able to identified two close-spaced Maca-specific whole genome duplications (WGDs, ~ 6.7 Mya) in genome assembly of Maca, which when compared with close-related *Brassicaceae* species revealed expansions of Maca genes and gene families involved in abiotic stress response, hormone signalling pathway and secondary metabolite biosynthesis via WGDs. Retention and subsequent evolution of many duplicated genes may account for the morphological and physiological changes in Maca adapting to high altitude environment (i.e. small leaf shape and self-pollination). Additionally, some duplicated Maca genes were identified with functions in morphological adaptation (i.e. MYB59) and development (i.e. GDPD5 and HDA9). The above study (49) may form the basis to an explanation how plants like *L. peruvianum* studied in the present and previous papers from this series (16, 17), acquired high altitude adaptation, which amongst multiple manifestations of the ongoing adaptation to the altitude where they grow, includes a variation in morphology of Maca hypocotyls in terms of colours, shapes and size.

Indicated by Jing Zhangi *et al.* (49) important roles of WGDs in adaptation of the Andean Maca to high altitude and switching-off the redundant pathways and networks are associated with a massive gene loss, rewiring and reorganization for the emergence of new functions as reported by De Smet and Van de Peer (50). The combined influences of environmental factors acting as a non-specific stress on the growing plants as experienced in hypocotyls of the four Maca phenotypes cultivated and harvested at increased altitudes a.s.l. being reported in the present study may explain inconsistency in obtained DNA spectra linked to both phenotypes and altitude of cultivation. Additional two month opened air drying in full sun (UV effect) with associated substantial changes in day-night temperature (inducing maceration of soft tissue in hypocotyls during repeated warming up and cooling down/freezing at night) as observed at the altitude where Maca was harvested may contribute to changes and observed inconsistency in DNA profiles in phenotypes of Maca reported for the same Maca phenotype collected from different cultivation altitudes.

The results presented in this paper could not confirm results reported by Chen *et al.* (48) who observed an existence of genetic variability sufficiently high to distinguish DNA Maca from the other species. Comparison of DNA spectra between Maca phenotypes was not included in Chan *et al* (48) study and similarly, comparison of the DNA spectra between Maca phenotypes and other plant species was not subject of the present study – hence, were not conducted and should be included in the further genetic study. In addition, there were too narrow representation of samples analysed in the present study in relation to Maca phenotypes originating from several cultivation locations. Therefore, no conclusive identification nor establishment of a reference for genetic profiles was possible to allow for the distinction of four Peruvian Maca phenotypes from their DNA profiles cultivated at various altitudes in the high Andes.

Despite the limited number of Maca phenotype samples used in the presented study, the obtained results suggest however, that phytochemical and differences in genetic profiles associated with Maca phenotypes and the altitude where Maca is cultivated, once properly standardised, have a real potential in becoming a routine procedure to determine authenticity of the marketed Maca products. It appears that more in-depth approach to the DNA Maca phenotypical genetic diversity is needed to rely on different loci to distinguish Maca phenotypes using own or other DNA sequence databases.

### Concluding Remarks

The results presented in this paper demonstrate distinct differences in phytochemical profiles and concentration of Glucosinolates adopted as the marker of Maca physiological activity in the four Maca phenotypes, induced by the altitude where they were grown and harvested before being used in commercial therapeutic formulations. The higher the altitude where Red and Purple Maca phenotype is grown the significantly higher Glucosinolates concentration was observed, while Black Maca showed reversed but also significant trend.

The results also indicate that in contrast to Maca grown at higher altitudes (at and above 4,200 m a.s.l.), the Maca cultivated at lower altitudes (at and below 4,000 m a.s.l.) may provide different therapeutic and health benefits, from Maca phenotypes sourced from an unknown cultivation location in terms of cultivation altitude when used in marketed products.

It is reasonable to assume that the altitude at which the four phenotypes of Peruvian Maca *L. peruvianum* are grown in High Andes, may be responsible for different physiological functionality. This aspect, which may be of importance in selecting the appropriate raw material for specific Maca preparations with an expected therapeutic functionality, warrants further confirmation in an *in vivo* laboratory and clinical study on humans.

There was a substantial genetic diversity in various coloured Maca phenotypes selected from disparate crops, various hypocotyl sizes and grown on a single plantation or in geographically distant locations, which confirm the observations made in a previously reported study. However, there were no clearly defined sequences in DNA bands within each location and altitude where Maca was cultivated, nor within each of the four compared phenotypes that could be characteristic enough to differentiate Maca grown at different altitudes on the basis of the DNA profile.

Although promising, insufficiently precise differences in DNA sequences failed to distinguish, without any reasonable doubt, four Maca phenotypes cultivated either in the same or geographically-distant locations, and harvested at different altitudes a.s.l. Further research on DNA sequences is needed, using both more specific primers and larger numbers of Maca phenotypes collected from various cultivation locations, taking into account biosynthesis of secondary metabolites and adaptation pathways induced by harsh environment at varied altitudes where Maca is cultivated in high Andes.
